# Maternal perceived stress and infant behavior during the COVID-19 pandemic

**DOI:** 10.1038/s41390-023-02748-2

**Published:** 2023-07-27

**Authors:** Holly Bradley, Dana Fine, Yasmin Minai, Laurel Gilabert, Kimberly Gregory, Lynne Smith, Wei Gao, Gina Giase, Sheila Krogh-Jespersen, Yudong Zhang, Lauren Wakschlag, Natalie H. Brito, Integra Feliciano, Moriah Thomason, Laura Cabral, Ashok Panigrahy, Alexandra Potter, Leigh-Anne Cioffredi, Beth A. Smith

**Affiliations:** 1 Developmental Behavioral Pediatrics, Children’s Hospital Los Angeles, Los Angeles, CA, USA.; 2 Cedars-Sinai Medical Center, Biomedical Imaging Research Institute, Los Angeles, CA, USA.; 3 Cedars-Sinai Medical Center, Department of Obstetrics and Gynecology, Los Angeles, CA, USA.; 4 Harbor-UCLA Medical Center Department of Pediatrics, David Geffen School of Medicine, Los Angeles, CA, USA.; 5 Department of Medical Social Sciences, Northwestern University, Chicago, IL, USA.; 6 Department of Applied Psychology, New York University, New York, NY, USA.; 7 Department of Child and Adolescent Psychiatry, New York University, New York, NY, USA.; 8 Department of Radiology, University of Pittsburgh Medical Center, Pittsburgh, PA, USA.; 9 University of Vermont Medical Center, Psychiatry, Burlington, VT, USA.; 10 Department of Pediatrics, University of Vermont Medical Center, Larner College of Medicine at the University of Vermont, Burlington, VT, USA.; 11 The Saban Research Institute, Children’s Hospital Los Angeles, Los Angeles, CA, USA.; 12 Department of Pediatrics, University of Southern California, Los Angeles, CA, USA.

## Abstract

**BACKGROUND:**

Maternal stress has negative consequences on infant behavioral development, and COVID-19 presented uniquely stressful situations to mothers of infants born during the pandemic. We hypothesized that mothers with higher levels of perceived stress during the pandemic would report higher levels of infant regulatory problems including crying and interrupted sleep patterns.

**METHODS:**

As part 6 sites of a longitudinal study, mothers of infants born during the pandemic completed the Perceived Stress Scale, the Brief Infant Sleep Questionnaire, and an Infant Crying survey at 6 (*n* = 433) and 12 (*n* = 344) months of infant age.

**RESULTS:**

Maternal perceived stress, which remained consistent at 6 and 12 months of infant age, was significantly positively correlated with time taken to settle infants. Although maternal perceived stress was not correlated with uninterrupted sleep length, time taken to put the infant to sleep was correlated. Perceived stress was also correlated with the amount of infant crying and fussiness reported at 6 months.

**CONCLUSIONS:**

Mothers who reported higher levels of perceived stress during the pandemic reported higher levels of regulatory problems, specifically at 6 months. Examining how varying levels of maternal stress and infant behaviors relate to overall infant developmental status over time is an important next step.

## INTRODUCTION

Coronavirus disease 2019 (COVID-19) is a highly infectious respiratory disease caused by severe acute respiratory syndrome coronavirus 2 (SARS-CoV-2) that began to rapidly spread worldwide in early 2020 and quickly became an unprecedented pandemic.^1^ Physical symptoms have been well studied and include fever, cough, fatigue and breathing difficulties, amongst others.^2^ In terms of psychological symptoms, it is suggested that anxiety, depression, post-traumatic stress disorder (PTSD) and distress are all associated with post-COVID-19 infection.^3^ The COVID-19 pandemic additionally presented a high level of psychological stress for individuals who were not directly infected. Noted increase in anxiety, depression, somatization, and PTSD was observed globally within the first few months of the pandemic, and this was exacerbated specifically in women under 40 years of age.^4,5^ The combination of COVID-19, pregnancy, and social isolation bears examining. Thus, in this paper, we will explore the mental health experiences of mothers who gave birth during the COVID-19 pandemic and the associations with their infant’s behaviors in their first year of life. This will allow us to more clearly understand these associations to work towards minimizing any long-term impacts of the pandemic if necessary.

Pregnant women and mothers of young children were disproportionately affected by the uniquely stressful situation of the COVID-19 pandemic.^4^ These stressors include, but were not limited to, financial strain,^6^ loss of childcare,^5^ food insecurity,^7^ an increase in domestic violence,^8^ worse pregnancy outcomes,^9^ and changes in medical care due to social distancing.^10^ In addition, mothers were significantly more involved in childcare during the pandemic,^11^ heightening their stress and exposure of that stress to their infant.^12^ When mothers perceive themselves to be stressed, they are more likely to be distant and less engaged with their child,^13^ which can lead to long term social and emotional issues.

Maternal stress can have consequences for infant development. It has been previously found that exposure to prenatal maternal stress is predictive of delays in cognitive,^14^ language,^15^ and motor^16^ development. Also, behavioral issues including poorer temperament,^17^ attentional processing,^18^ behavioral disinhibition, and stress regulation^19^ throughout infancy and childhood are related to increased maternal stress during pregnancy. The current state of knowledge regarding the associations between maternal stress, and consequent infant developmental outcomes during the COVID-19 pandemic is limited. It has been reported that prenatal maternal stress predicted decreased surgency in 3-month-old infants born during the COVID-19 pandemic,^20^ and predicted infant temperament, with mothers reporting higher levels and greater lability of perceived stress also reporting lower levels of infant regulation at 4 and 6 months of age.^21^ Other recent research has found that symptoms associated with maternal depression were not related to worse infant temperament outcomes^22^ and that there is not an association between pandemic exposure and infant developmental outcomes.^23^ These findings present contradictory results regarding potential associations between COVID-19 related maternal stress and infant behaviors. To contribute to these findings, this current study focused on the associations between maternal stress and two specific infant behaviors during COVID-19: sleeping and crying.

Sleeping difficulties and excessive crying in infancy are two regulatory problems that are known risk factors for emotional and behavioral issues in later childhood.^24^ Established sleep cycles play a crucially important role in infant development, namely growth and healing, emotion processing, and regulating hormone and immune function, amongst others.^25^ Adequate sleep is associated with a reduced risk of long-term behavioral and emotional problems, as well as improved mood and well-being.^26^ Studies have shown that maternal stress, both during and after pregnancy, is closely associated with infant sleep disorders^27,28^ with maternal stress during pregnancy altering fetal corticocerebellar connectivity causing an increase in sleep problems post-birth.^27^ A lack of quality sleep can hinder infant development in different domains including language, memory, executive function, and overall cognitive development.^29^ Further, crying is an important behavior that contributes to an infant’s healthy psychosocial development. Excessive crying beyond the first 3 months of life may be associated with regulatory issues, have a negative effect on the infant-mother relationship,^30^ and contribute to behavioral and mental problems later in life.^31^ Maternal stress has been shown to be linked with excessive infant crying and fussiness,^32,33^ with infants of mothers who self-reported high scores of negative life changes exhibiting increased crying and fussiness, specifically within the first 6 months of life. Excessive crying and fussiness in the first year of life has been shown to double the risk of hyperactivity, mood, and behavioral problems at the age of 5 years old.^34^

In this study, we analyzed maternal surveys taken at infant ages 6 and 12 months in women living in varied regions of the United States. Surveys addressed maternal perceived postnatal stress, infant crying, and infant sleeping habits. This research was conducted a part of the larger COVID-19 and Perinatal Experiences (COPE) study designed to assess the feelings and experiences of pregnant women and mothers during the COVID-19 pandemic. The aim of this study was to test the hypothesis that mothers of infants born during the pandemic who reported higher levels of perceived postnatal stress during the COVID-19 pandemic would also report higher levels of regulatory problems including crying and interrupted sleep patterns. To do this, our objectives in this study were to test for associations between maternal perceived stress and infant regulatory problems at 6 months, 12 months, and averaged across 6 and 12 months of age to establish if this association was persistent across these two time points.

## METHODS

### Participants and recruitment

This was a parallel study taking place at 6 different sites in the USA: Children’s Hospital Los Angeles (CHLA), Cedars Sinai Medical Center, New York University (NYU), The University of Vermont (UVM), Children’s Hospital of Pittsburgh (CHP), and Northwestern University. Each site obtained approval from their respective Institutional Review Boards and conducted independent recruitment and data collection using the same study design. The de-identified data were shared from sites to CHLA for aggregate analyses. The inclusion criteria were any pregnant person at any gestational age, or any child and mother dyad when the child is birth to 6 months old. There were no exclusion criteria. Pregnant individuals, or mothers who gave birth within the last 6 months, were recruited via flyers and social media, or from existing studies. Once consented, the surveys were sent and completed through REDCap throughout 2021 and early 2022. Participants were compensated for each group of surveys they completed in the form of an electronic gift card. Mothers completed the surveys within 1 month of their infant being 6 and/or 12 months old. Only complete surveys met criteria for inclusion. A total of 433 surveys were included at 6 months (CHLA *n* = 43, Cedars Sinai Medical Center *n* = 115, NYU *n* = 160, UVM *n* = 55, CHP = 2, Northwestern = 58), and 344 at 12 months (CHLA *n* = 41, Cedars Sinai Medical Center *n* = 87, NYU *n* = 121, UVM *n* = 55, CHP = 2, Northwestern *n* = 38). The demographic characteristics of our sample of infants are shown in Table 1.

### Materials

The three surveys of interest in this research study were administered to mothers amongst a battery of questionnaires assessing child negative affect, developmental milestones, parenting stress, maternal mental health, and maternal physical health. The surveys were completed online via REDCap. The three surveys analyzed here were the Perceived Stress Scale (PSS), the Brief Infant Sleep Questionnaire (BISQ), and an Infant Crying Survey.

#### Perceived stress scale (PSS).

The PSS^35^ is a widely used psychological screening tool to measure an individual’s perception of self-stress. Questions on the PSS are general in nature and not content specific, and so can be applied across population groups. The original PSS consisted of 14 items but is most frequently used today as a 10-item scale, as in this study. The PSS is a self-report questionnaire made up of items that are designed to assess how unpredictable, uncontrollable, and overwhelming an individual’s current life circumstances have been during the last month. Results on the PSS can range from 0 to 40, with higher scores indicating higher levels of perceived stress: 0–13 is considered low stress, 14–26 is considered moderate stress, and 27–40 is considered to be high stress. The PSS score of each mother is used as the variable in this research study to assess maternal perceived stress.

#### The brief infant sleep questionnaire (BISQ).

The BISQ^36^ is a parent-reported questionnaire used to assess infant and toddler sleep patterns and sleep-related behaviors over the past week. The BISQ is compiled of 33 questions that are split into three sections: nocturnal sleep duration, night waking, and method of falling asleep. Scores on the BISQ are from 0–100; higher scores indicate better sleep quality and a more positive perception from the parent on their infant’s sleep. In this study, the BISQ was not scored in full. Instead, we focused on two questions of interest : (1) *How long does it take to put your baby to sleep in the evening (in hours and minutes)?* (2) *Approximately how many hours is your infant*’*s longest sleep period during the day or night?* As opposed to using the summary scores for analysis, we are interested in investigating specific infant sleep behaviors. These two questions were chosen a priori and allowed us to consider how long it takes to settle the infant, and what is the infant’s longest uninterrupted sleep period, allowing us to obtain an overview of their typical sleep behaviors and gauge any potentially disruptive patterns, best capturing the behaviors of interest in this study. These two questions allowed us to obtain two sleep variables for this present study: ‘longest period of uninterrupted sleep’ and ‘time taken to settle infant’. The variable ‘longest period of uninterrupted sleep’ was scored in hours and minutes, and ‘time taken to settle infant’ was scored in hours.

#### Infant crying survey.

The infant crying survey consisted of 10 questions about crying durations and parental perceptions of their infants crying. The question of interest in this research study is *How often does your baby fuss or cry in general?*, this allowed us to see if the mother perceived their infant to cry: (1) not at all, (2) a little, (3) much, (4) very much (with the option of a decline to answer). Consistent with the sleep questions, we were not interested in the summary scores of the survey for analysis, but interested in investigating a specific behavior. This question was chosen as it allows us to gauge the mother’s perception of their infant’s level of crying and fussiness. This question allowed us to obtain the ‘crying’ variable for this research study and was rated on a scale of 1–4.

### Data analysis

The goal of this study was to establish the associations between maternal perceived stress during the COVID-19 pandemic and infant behaviors. Thus, data from the 6 sites (CHLA, Cedars Sinai Medical Center, NYU, UVM, CHP, and Northwestern) were aggregated into one group analysis in this manuscript. The data were split into two groups: 6-month time point data (*n* = 433) and 12-month time point data (*n* = 344). SPSS (IBM SPSS Statistics, version 28) was used to perform all statistical analyses. Data were analyzed at 6 months, 12 months, and averaged across 6 and 12 months to investigate three research questions: (1) What is the relationship between maternal perceived stress and the amount of time taken to settle the infant? (2) What is the relationship between maternal perceived stress and longest period of uninterrupted sleep?, and (3) What is the relationship between maternal perceived stress and the amount of infant crying and fussiness? Data were checked for normality with Shapiro–Wilk tests of normality. As the data were normally distributed, we reported descriptive statistics for each variable: range, mean (M) and standard deviation (SD). For research question number one, we conducted Pearson product-moment correlations to test for associations between maternal PSS scores and the time taken (in hours and minutes) to settle the infant at 6 month, 12 months, and averaged across the two time points. A multiple linear regression was undertaken to test if maternal PSS Scores, longest uninterrupted sleep, or infant crying/fussiness significantly predicted time taken to settle the infant across 6 and 12 months. For research question number two, we conducted Pearson product-moment correlations to test for associations between maternal PSS scores and the longest period of uninterrupted sleep at 6 month, 12 months, and averaged across the two time points. A multiple linear regression was undertaken to test if maternal PSS Scores, time taken to settle the infant, or infant crying/fussiness significantly predicted time taken to settle the infant across 6 and 12 months. For research question number three, we conducted Pearson product-moment correlations to test for associations between maternal PSS scores and the amount of infant crying and fussiness at 6 month, 12 months, and averaged across the two time points. A multiple linear regression was undertaken to test if maternal PSS Scores, longest uninterrupted sleep, or time taken to settle the infant significantly predicted time taken to settle the infant across 6 and 12 months. For all analyses, significance was set at *p* < 0.05

## RESULTS

### Maternal perceived stress

**Fig. 1:**
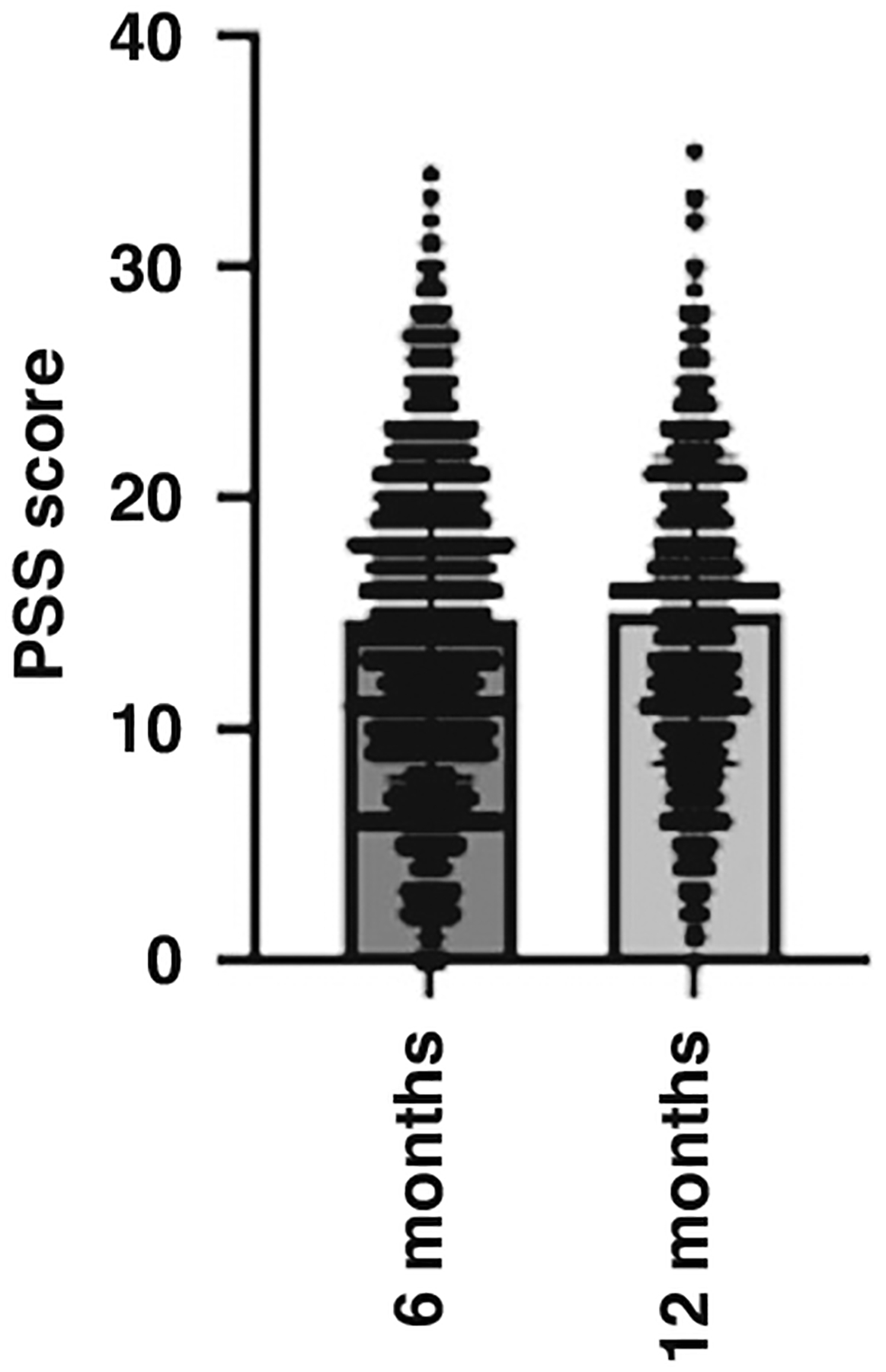
A scatter plot with bar to illustrate the mean and range of maternal PSS scores at 6 and 12 months. The horizontal lines represent the total range of scores and within this, the number of responses at each score. The large bar represents the mean of scores.

When infants were 6 months of age, scores on the PSS ranged from 0 to 34 (M = 14.7, SD = 7.1). When infants were 12 months of age, scores on the PSS ranged from 0 to 35 (M = 15, SD = 6.9). Figure 1 illustrates the PSS mean scores and ranges at 6 and 12 months. Averaged across 6 and 12 months, the mean score for the PSS was 14.9. This shows that maternal perceived stress, on average, remained consistent at 6 and 12 months, and falls into the category of moderate stress as per the PSS scoring guidelines. For individuals who had data at both 6 and 12 months, we calculated the change in PSS scores between the two time points. The average change in PSS scores between 6 and 12 months was +0.3 points.

### Infant sleeping

The two variables of interest are: (1) Time taken to settle infant, (2) Longest uninterrupted sleep period.

#### Time taken to settle infant.

**Fig. 2:**
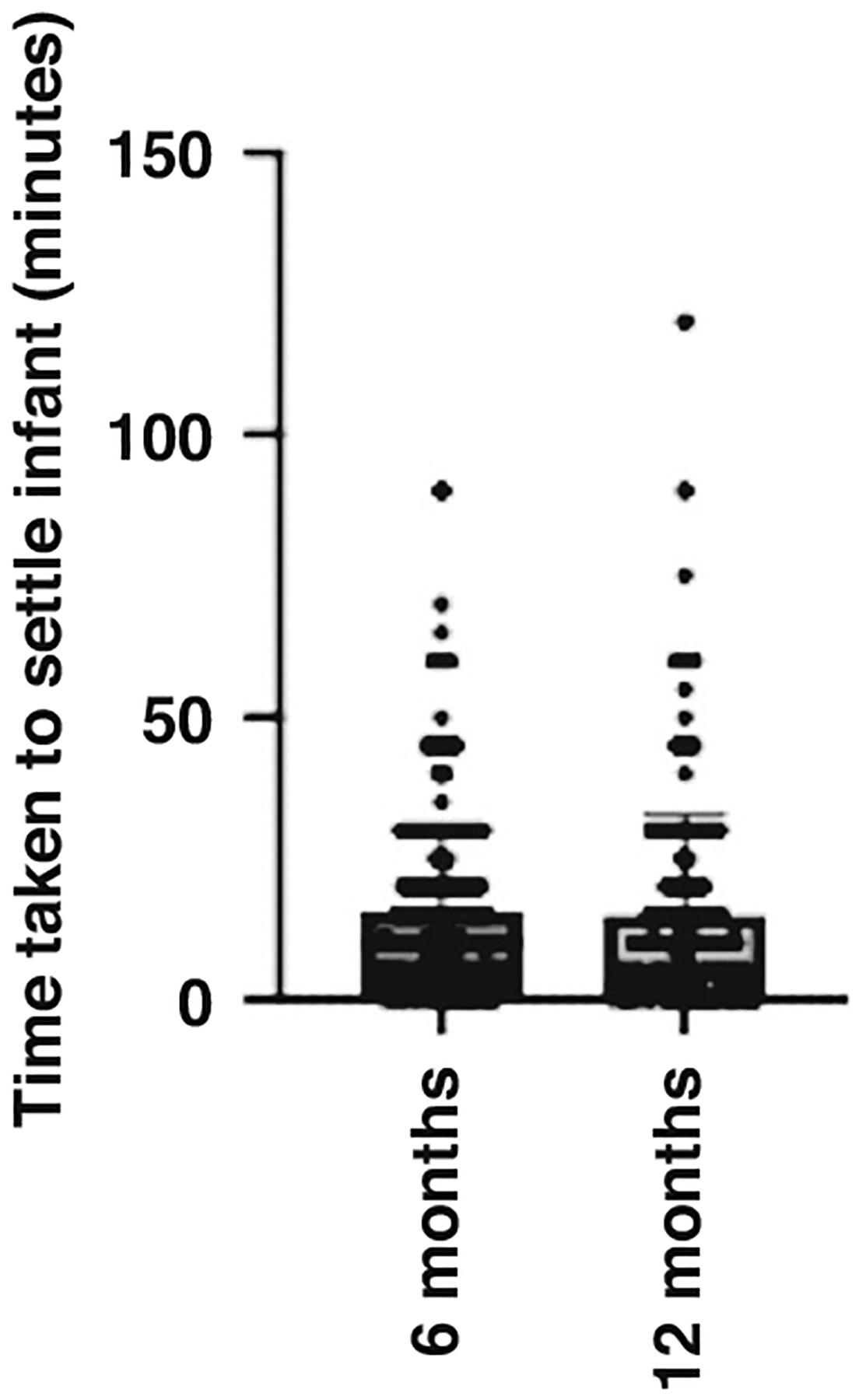
A scatter plot with bar to illustrate the time taken to settle infant to sleep at 6 months and 12 months of age. The horizontal lines represent the total range of scores and within this. The number of responses at each score. The large bar represents the mean of scores.

**Fig. 3:**
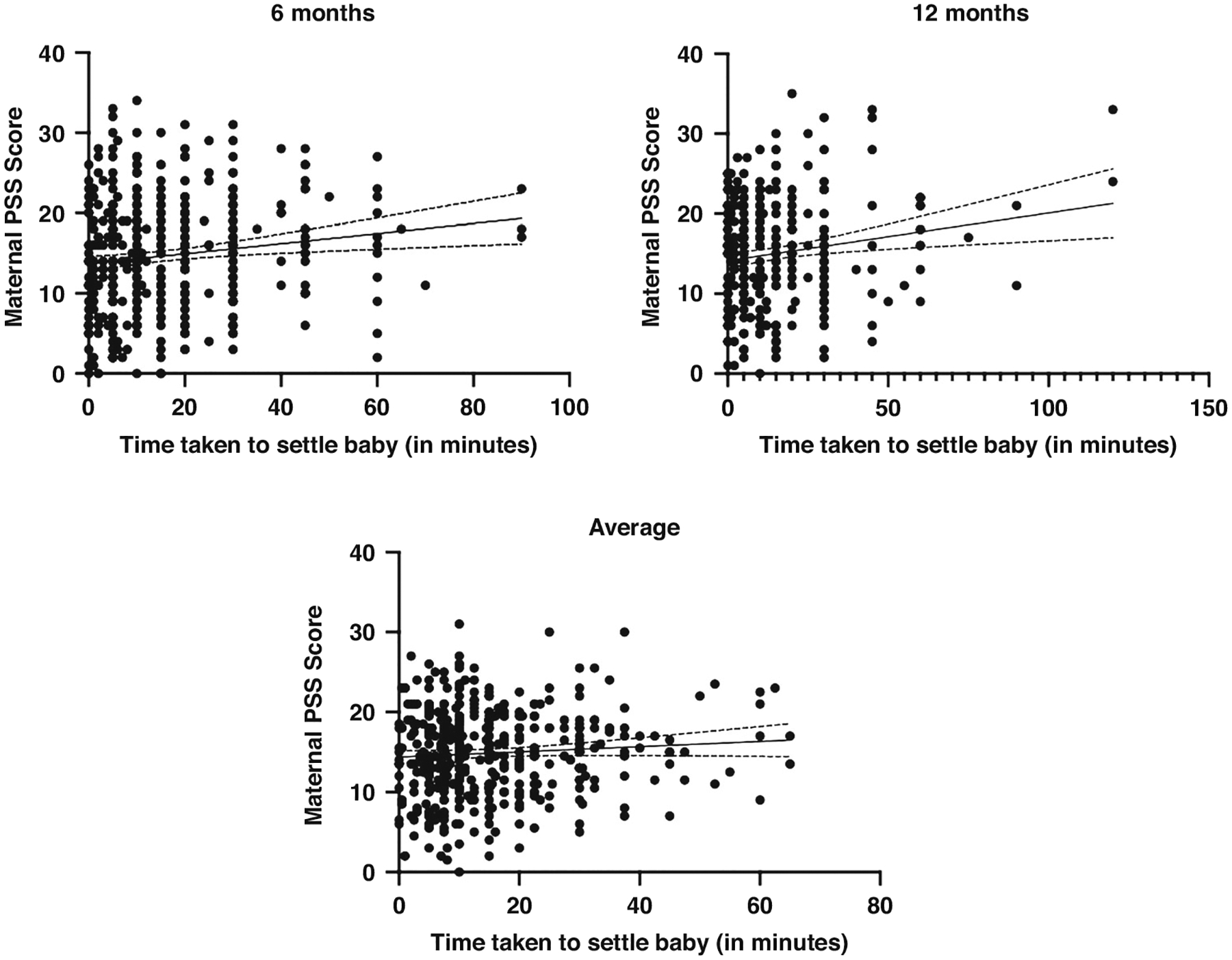
Scatter plots to illustrate the positive correlations between maternal PSS score and time taken to settle baby at 6 months, 12 months, and averaged across 6 and 12 months. The scatter plots include a regression line with confidence intervals.

At 6 months of age, the time taken to settle the infant ranged from 0–90 min (M = 16.1, SD = 17.8). At 12 months of age, the time taken to settle the infant ranged from 0–120 min (M = 13.1, SD = 16.5). See Fig. 2 for an illustration of the range and mean time taken to settle the infant at 6 and 12 months old. Averaged across 6 and 12 months, the time taken to settle the infant was 15.4 min. Maternal perceived stress was significantly positively correlated with the time taken to settle the infant at 6 months old (*r* = 0.162, *p* < 0.001, 95% CI [0.068–0.252]) and 12 months old (*r* = 0.141, *p* = 0.009, 95% CI [0.036–0.243), these results indicate a low to moderate correlation. When averaged across 6 and 12 months, maternal perceived stress had a low to moderate correlation with time taken to settle the infant (*r* = 0.113, *p* = 0.018, 95% CI [0.019–0.205]). The more stressed the mother perceived herself to be, the longer it took for the infant to be settled at bedtime. See Fig. 3 for a visual representation of these three significant, positive correlations. A multiple linear regression was used to test if any of the variables (maternal PSS Scores, longest uninterrupted sleep, and infant crying/fussiness) significantly predicted time taken to settle the infant across 6 and 12 months. The overall regression was statistically significant (R^2^ = 0.189, F(432, 429) = 5.283, *p* = 0.001, 95% CI [11.459–34.652]). It was found that maternal perceived stress significantly predicted time taken to settle the infant (β = −252, *p* = 0.021), as did the longest period of uninterrupted sleep (β = −1.369, *p* < 0.001); the longer the infant slept, the longer it took them to settle.

#### Longest uninterrupted sleep.

At 6 months of age, the longest uninterrupted sleep ranged from 3.5–12.5 h (M = 10.1, SD = 1.5). At 12 months of age, the longest uninterrupted sleep ranged from 4–15 h (M = 10.5, SD = 1.5). Across 6 and 12 months, the longest period of uninterrupted sleep averaged 10.2 h.

**Fig. 4:**
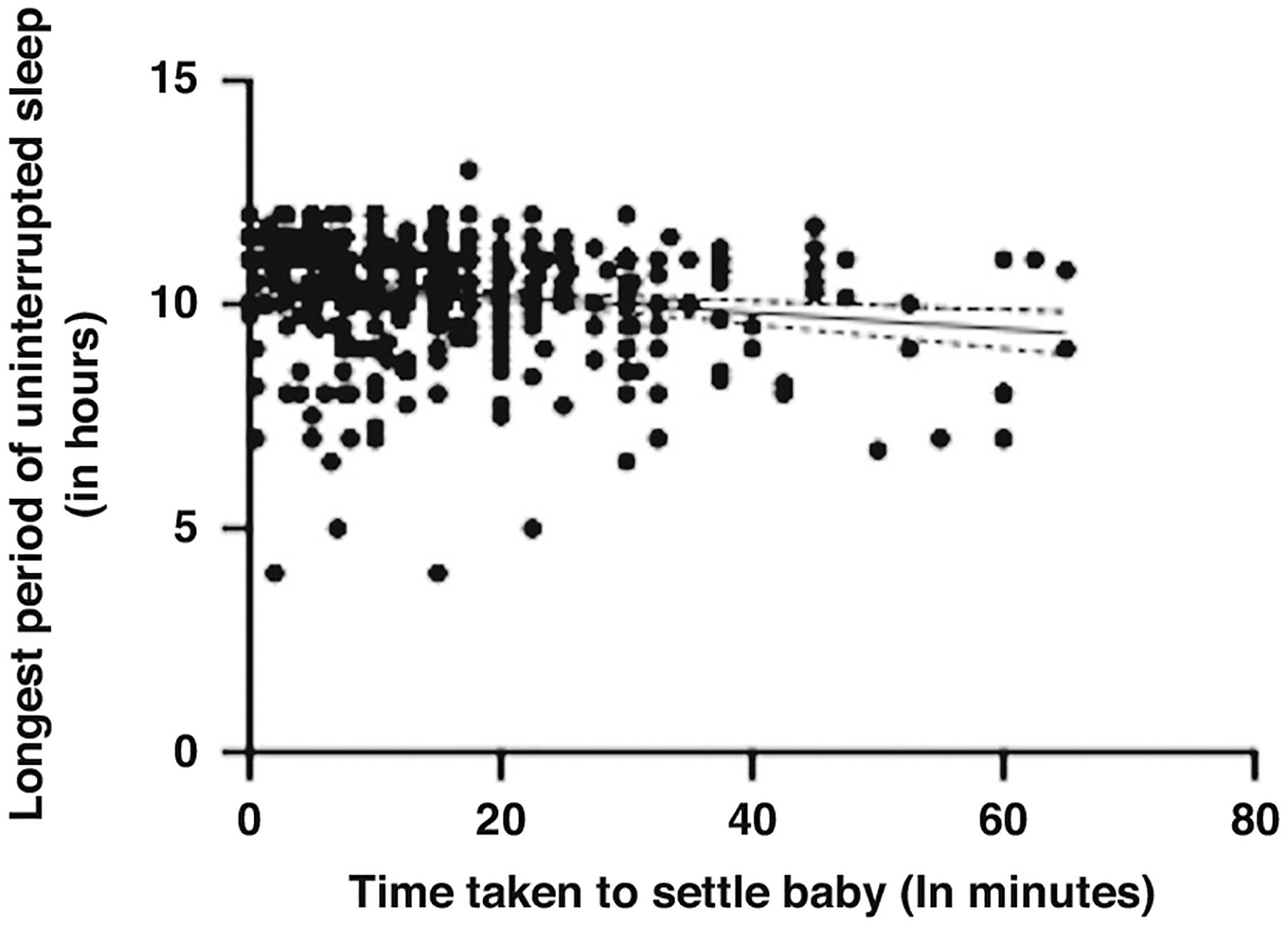
A scatter plot to show the relationship between longest period of uninterrupted sleep and time taken to settle the infant averaged across 6 and 12 months. The scatter plots include a regression line with confidence intervals.

Maternal perceived stress was not significantly correlated with the longest period of uninterrupted sleep at 6 months old (*r* = −0.029, *p* = 0.550, 95% CI [−0.123–0.066]) or 12 months old (*r* = −0.036, *p* = 0.511, 95% CI [−0.141–0.070]). When averaged across 6 and 12 months, maternal perceived stress was not significantly correlated with the infant’s longest uninterrupted sleep period (*r* = −0.005, *p* = 0.914, CI [−0.099–0.089]). A multiple linear regression was used to test if any of the variables (maternal PSS Scores, time taken to settle infant, and infant crying/fussiness) significantly predicted the longest period of infant uninterrupted sleep across 6 and 12 months. The overall regression was statistically significant (R^2^ = 0.164, F(432, 429) = 3.939, *p* = 0.009, 95% CI [10.203–11.604]). The only variable to significantly predict the longest period of uninterrupted sleep was the time taken to settle the infant (β = −0.015, *p* = 0.003). Time taken to put infant to sleep, and longest uninterrupted sleep had a low to moderate significant negative correlation (*r* = −0.147, *p* = 0.002, 95% CI [−0.238–0.054]). See Fig. 4 for a visual representation of the relationship between these two variables. This means that the longer time taken to settle the infant was predictive of shorter periods of uninterrupted sleep, and longer periods of uninterrupted sleep were predictive of less time taken to settle the infant. As the two sleep variables were strongly correlated, the effect of maternal perceived stress on one variable was likely to indirectly affect the other. Thus, as maternal stress at 6 and 12 months predicted longer times to settle the infant, this may have also related to shorter periods of uninterrupted sleep in these infants, potentially leading to an overall worse quality of sleep.

### Infant crying

Mothers were asked to report how often their infant fusses or cries with the potential answers being (1) not at all, (2) a little, (3) much, (4) very much. At 6 months of age, answers ranged from 1–4 (M = 2, SD = 0.4). The distribution of answers was as follows: not at all = 34, a little = 369, much = 22, very much = 7 (with 1 parent declining to answer). At 12 months of age, answers ranged from 1–4 (M = 1.9, SD = 0.4). The distribution of answers was as follows: not at all = 30, a little = 290, much = 17, very much = 6 (with 1 parent declining to answer). Averaged across 6 and 12 months, the amount of reported fussing and crying was 1.9.

**Fig. 5:**
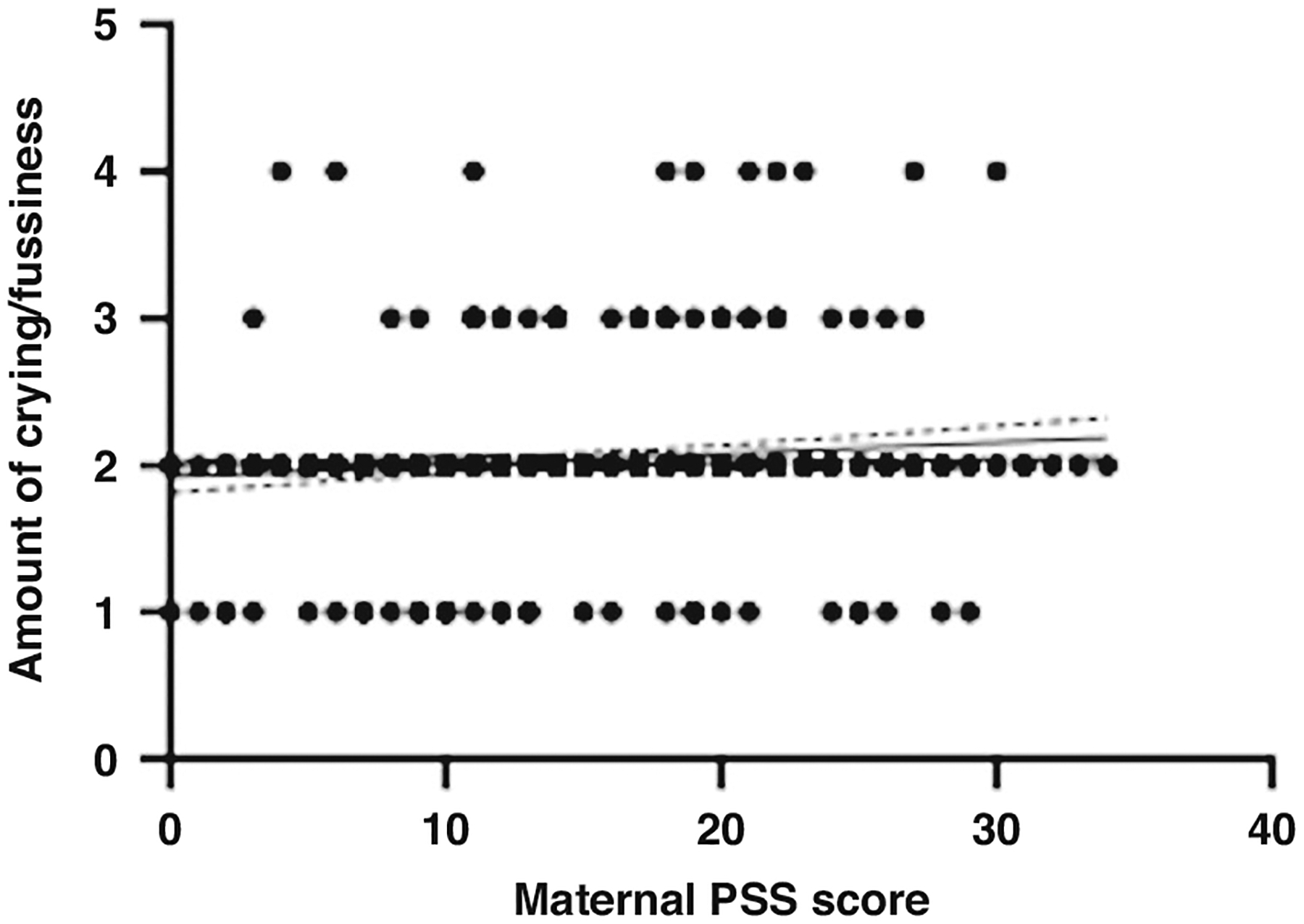
A scatter plot to show the relationship between maternal perceived stress scores and the amount of infant fussiness and crying (from 1 ‘not at all’ to 4 ‘very much’) at 6 months old. The scatter plots include a regression line with confidence intervals.

Maternal stress had a low to moderate correlation with the amount of crying and fussiness *(r* = 0.112, *p* = 0.020, 95% CI [0.020–0.200]) at 6 months of age, with mothers reporting higher levels of perceived stress also reporting higher levels of infant crying and fussiness (Fig. 5). Maternal PSS scores were not correlated with infant crying and fussiness at 12 months of age. When averaged across 6 and 12 months, maternal perceived stress was not significantly correlated with infant’s crying and fussiness (*r* = 0.080, *p* = 0.096). A multiple linear regression was used to test if any of the variables (maternal PSS scores, time taken to settle infant, and longest interrupted sleep period) significantly predicted the amount of infant crying and fussiness across 6 and 12 months. The overall regression model was not statistically significant (R^2^ = 0.024, F(432, 429) = 1.958, *p* = 120, 95% CI [1.797–2.437]).

## DISCUSSION

This study investigated the associations between maternal perceived stress and infant behavior, specifically sleeping and crying, in the infant’s first year of life (at 6 and 12 months old), during the COVID-19 pandemic. Our results showed that maternal perceived stress, on average, did not change between infant age of 6 and 12 months, illustrating that maternal stress, as measured by the PSS, was consistently reported as ‘moderate stress’ across this time period. In a study of 900 mothers, it was found that depression and anxiety increased during the pandemic compared to before.^37^ Further, it was found in 2020 that parents to young children who were exposed to a greater number of COVID-19 related stressors also experienced higher levels of perceived stress.^38^ Thus, although we do not have a record of pre-pandemic data of PSS scores for the mothers, we can assume that the pandemic caused this to be heightened.

There was a positive and significant correlation between maternal PSS scores and time taken to settle the infant; mothers who reported higher levels of perceived stress also reported longer times to settle their infant at night at both 6 and 12 months. Although maternal stress was not found to be correlated with periods of uninterrupted sleep at any time point, time taken to put the infant to sleep was strongly negatively correlated with the longest period of uninterrupted sleep at 6 and 12 months. As maternal perceived stress predicted longer times to settle the infant, one interpretation of the results is that time taken to settle the infant may relate to shorter periods of uninterrupted sleep in these infants, potentially leading to an overall worse quality of sleep. Finally, maternal stress was positively significantly correlated with the amount of infant crying and fussiness at 6 months of age, but not at 12 months. Our results identified early associations between maternal perceived stress during the pandemic and reported infant behavior, specifically sleeping, and crying, highlighting potential developmental risks for infants growing up during the pandemic.

With regards to maternal mental health, we showed that maternal perceived stress remained consistently classified as ‘moderate’ over the first year of life; we do not have national data for pregnancy to compare pre-COVID. This adds to the existing literature that COVID-19 presented a uniquely stressful situation to mothers of infants born during the pandemic^4^ and shows that this stress did not change across a six-month period. Pandemic-related stressors, including financial pressure,^6^ physical distancing and a strain on childcare,^5^ amongst others, especially affected mothers and predisposed them to moderate stress, as illustrated in this study. With regards to the link between maternal stress and infant behavior, our findings fall in line with existing literature illustrating that negative maternal experiences are associated with more potentially disruptive infant behaviors.^20,21^ The first 1000 days of human life are a developmentally sensitive window for stress exposure in which the brain grows more quickly than at any other time point in an individual’s life.^39^ Stress has already been linked to adverse child and maternal outcomes, however, the effects of maternal stress on infant behavior during the first 1000 days warrants further investigation as it provides information about where to consider intervening. It is more important than ever to further understand how prenatal and postnatal stress might affect infant behavior during the first 1000 days of life, as stress related to the pandemic will impact the next generation.^40^ Our findings illustrate that maternal perceived stress was constant at 6 and 12 months of infant age, and the mothers who reported higher levels of stress during the pandemic also reported higher levels of potentially disruptive infant behavioral habits, specifically at 6 months of age. These findings highlight the need for increased maternal support in the first year of life.

Limitations of this study should be noted. Because we did not collect pre-pandemic data from these mothers, we are not able to determine if perceived stress levels were changed as a result of the COVID-19 pandemic. We do not have information about people who did not participate in the study, or who participated but did not complete these specific survey questions. Further, data presented here are based on maternal report survey data, and thus are subjective data; it will be important for future work to build upon these findings, interrogating infant behavioral and health correlates of increased parental stress in the context of the pandemic. Another limitation of the current study is that the validity of the measures obtained may be questioned due to the authors’ decision to not use the summary scores of the crying and sleeping questionnaires. However, looking at the single chosen items allows for a more nuanced understanding of the two constructs, sleeping and crying, being measured. We believe that the chosen items allowed us to investigate key sleeping and crying behaviors of interest to development. Finally, there are likely other confounding variables affecting maternal perceived stress and infant behavior that were not accounted for in this study such as maternal depression which is often used as an example of maternal stress, but is a separate construct. In addition, as maternal perceived stress was measured concurrently with infant regulatory problems, causal pathways cannot be inferred. For example, infants who are poorer sleepers are more likely to have tired and stressed mothers.

To summarize, our results support, in line with the hypothesis, that mothers who reported higher levels of perceived stress during the pandemic also reported higher levels of regulatory problems in their infants. Our study is an important first step in identifying correlates of maternal perceived stress during the pandemic and infant regulatory problems. Future work that enables testing of potential causal pathways will be useful. How varying levels of maternal stress and infant behaviors relate to overall infant developmental status in our study is unknown, future research should investigate the longitudinal effects of the pandemic on child developmental outcomes using methods that can examine bidirectional pathways and disentangle pandemic related vs. general stress in these pathways.

## Figures and Tables

**Table 1.** A table to show the demographic characteristics of the infants in the study.

	Demographic Characteristic	*N* (%)
Sex	Male	231 (53.3)
	Female	202 (46.7)
Ethnicity	Hispanic or Latino	83 (19.2)
	Not Hispanic or Latino	295 (68.1)
	Unknown	55 (12.7)
Race	American Indian or Alaska Native	8 (1.8)
	Asian	87 (20.1)
	Black or African American	55 (12.7)
	Native Hawaiian or Other Pacific Islander	3 (0.7)
	White	187 (43.2)
	Other	39 (9)
	Unknown	54 (12.5)

## Data Availability

The datasets used and/or analyzed during the current study available from the corresponding author on reasonable request.

## IMPACT:

Women giving birth during the COVID-19 pandemic who reported higher levels of stress on the Perceived Stress Scale also reported higher levels of infant fussiness and crying at 6 months old, and more disruptive sleep patterns in their infants at 6 months and 12 months old. Sleeping problems and excessive crying in infancy are two regulatory problems that are known risk factors for emotional and behavioral issues in later childhood. This paper is one of the first studies highlighting the associations between maternal stress and infant behaviors during the COVID-19 pandemic.
